# Phytochemical Profile of *Capsicum annuum* L. cv Senise, Incorporation into Liposomes, and Evaluation of Cellular Antioxidant Activity

**DOI:** 10.3390/antiox9050428

**Published:** 2020-05-15

**Authors:** Chiara Sinisgalli, Immacolata Faraone, Antonio Vassallo, Carla Caddeo, Faustino Bisaccia, Maria Francesca Armentano, Luigi Milella, Angela Ostuni

**Affiliations:** 1 Department of Science, University of Basilicata, via dell’Ateneo Lucano 10, 85100 Potenza, Italy; chiara.sinisgalli@unibas.it (C.S.); immacolata.faraone@unibas.it (I.F.); faustino.bisaccia@unibas.it (F.B.); mariafrancesca.armentano@unibas.it (M.F.A.); angela.ostuni@unibas.it (A.O.); 2 Spinoff Bioactiplant, via dell’Ateneo Lucano 10, 85100 Potenza, Italy; 3 Department of Science della Vita e dell’Ambiente, Sezione di Scienze del Farmaco, University of Cagliari, Via Ospedale 72, 09124 Cagliari, Italy; caddeoc@unica.it

**Keywords:** *Capsicum annuum* L., Senise cultivar, dried pepper, polyphenols, liposomes, oxidative stress, cellular antioxidant activity

## Abstract

Overproduction of oxidants in the human body is responsible for oxidative stress, which is associated with several diseases. High intake of vegetables and fruits can reduce the risk of chronic diseases, as they are sources of bioactive compounds capable of contrasting the free radical effects involved in cancer, obesity, diabetes, and neurodegenerative and cardiovascular diseases. *Capsicum annuum* L. cv Senise is a sweet pepper that is grown in the Basilicata region (Italy). It is an important source of polyphenols, carotenoids, and capsinoids and can play a key role in human health. In this study, an ethanol extract was obtained from *C. annuum* dried peppers and the analysis of the phytochemical composition was performed by LC-ESI/LTQ Orbitrap/MS. The extract was incorporated into liposomes, which showed small size (~80 nm), good homogeneity, negative surface charge, and good stability in storage. The biological activity of the extract was evaluated in the human hepatoma (HepG2) cell line, used as model cells. The extract showed no cytotoxic activity and reduced the intracellular reactive oxygen species (ROS) level in stressed cells. The antioxidant activity was further improved when the extract was loaded into liposomes. Moreover, the extract promoted the expression of endogenous antioxidants, such as catalase, superoxide dismutase, and glutathione peroxidase through the Nrf-2 pathway evaluated by RT-PCR.

## 1. Introduction

Every day, several factors, such as pollution, cigarette smoking, drugs, physical inactivity, and excessive alcohol consumption, can increase the production of free radicals and alter the homeostasis of the body [1]. The increase in free radicals, resulting in oxidative stress, is implicated in the etiology of several major human ailments, including cancer, cardiovascular diseases, neural disorders, diabetes, and arthritis.

Many studies have reported that a high intake of fruits, spices, and vegetables rich in polyphenols is linked to lower risk of chronic and degenerative diseases [2,3]. The biological activities of polyphenols have been attributed to their chemical structures, which make these compounds good electron or hydrogen atom donors capable of neutralizing free radicals [3] and thus restoring redox homeostasis. Moreover, they promote the expression of antioxidant enzymes involved in organism defenses such as catalase (CAT), superoxide dismutase (SOD), and glutathione peroxidase (GPX) [4,5].

Despite their health-promoting properties, polyphenols have a weak oral bioavailability due to their low water solubility, poor absorption, and rapid metabolism [5]. These problems can be tackled by using different drug delivery approaches, which can enhance polyphenols bioavailability and thus their therapeutic efficacy. Liposomes represent an optimal delivery system due to the morphological similarity with cell membranes and the ability to entrap both lipophilic and hydrophilic compounds that should be delivered to a specific target site [6].

*Capsicum annuum* L. cultivar Senise (Solanaceae) is a sweet pepper cultivated in the Basilicata region (Italy). Traditionally, it is sun-dried and eaten fried (“cruschi peppers”) or powdered and used as a spice. The “red gold of the Basilicata region”, as it is called for its similarity with saffron, is a very precious and important source of health-promoting compounds. Until now, few studies have investigated the biological activity and chemical composition of this pepper cultivar [7,8]. Particularly, they reported the antioxidant activity and potential hypoglycemic activity in vitro [7], which were ascribed to the presence of health-promoting compounds, such as carotenoids, polyphenols, terpenes, and ascorbic acid [7,8,9].

The aim of this study was to investigate the phytochemical profile of an ethanol extract of *Capsicum annuum* L. cultivar Senise and its antioxidant activity on cells. The HepG2 cell line was used as a model in the present work because its cells show many of the specialized functions that characterize human hepatocytes and they live longer than primary hepatocytes, improving the reproducibility [10,11]. The advantage provided by liposomal incorporation was evaluated. Furthermore, the molecular signaling pathways involved in the antioxidant activity of the extract were assessed. As far as we are aware, this is the first study that reports the biological activity of *C. annuum* extract in cells or incorporated in a vesicular carrier system.

## 2. Materials and Methods

### 2.1. Chemicals

Absolute ethanol, sodium phosphate monobasic (NaH_2_PO_4_), fluorescein, 2,2′-azobis(2-amidinopropane)dihydrochloride (AAPH), Dulbecco’s Modified Eagle Medium (DMEM), dimethyl sulfoxide (DMSO), [3-(4,5-dimethyl-2-thiazolyl)-2,5-diphenyl-2H-tetrazolium bromide] (MTT), 2′,7′-dichlorodihydrofluorescein diacetate (DCFH-DA), *N*-acetyl-*l*-cysteine (NAC), and *tert*-butyl hydroperoxide (*t*-BuOOH) were purchased from Sigma Aldrich S.p.A. (Milan, Italy). Trypsin-EDTA solution, fetal bovin serum (FBS), glutamine, penicillin-streptomycin, and phosphate saline buffer (PBS) were purchased from Euroclone (Milan, Italy). Reagents used for RT-PCR were purchased from Euroclone (Milan, Italy). Solvents used for LC-ESI/LTQOrbitrap/MS extraction and water were purchased from VWR (Milan, Italy), while acetonitrile and formic acid were purchased from Merck (Merck KGaF, Darmstadt, Germany).

Phospholipon 90G (>90% phosphatidylcholine; P90G) was purchased from Lipoid GmbH (Ludwigshafen, Germany).

### 2.2. Extract Preparation

Sun-dried red peppers were kindly provided by “Azienda agricola Casata del Lago” (Senise, Potenza, Italy; 40°08′35.1″ N 16°18′55.3″ E) during October 2016. Dried fruits (500 g) without seeds and petiole were cut into small pieces and extracted by maceration with absolute ethanol (1.2 L) in the dark at room temperature for 48 h. The extraction procedure was repeated three times. *C. annuum* extract (CAE) (yield of 11.70% *w*/*w*) was filtered with filter paper and dried by means of a rotary evaporator (IKA RV 10). Then, it was stored in the dark at room temperature.

### 2.3. LC-ESI/LTQOrbitrap/MS

The phytochemical characterization of CAE was carried out by an in-house HPLC method coupled with a mass spectrometer, which associates the linear trap quadrupole with an OrbiTrap mass analyzer. LC-ESI/LTQOrbitrap/MS analyses were performed in positive and negative ion modes using an Accela 600 HPLC system (Thermo Scientific, Bremen, Germany) coupled to an LTQ OrbiTrap XL mass spectrometer (Thermo Scientific, Bremen, Germany). Separation was achieved using a Luna 2.5 μm C18 (100 mm × 2.10 mm) column (Phenomenex, Aschaffenburg, Germany).

The employed mobile phases were water + 0.1% formic acid (solvent A) and acetonitrile (solvent B). The flow rate was 0.2 mL/min, and the gradient was as follows: 2% of B at 0 min until 1 min, 40% at 21 min, 95% at 22 min until 25 min, returning to 2% of B at 26 min until 35 min.

MS settings were as follows: in positive ion mode, source voltage 3 kV, capillary voltage 49 V, tube lens voltage 120 V; in negative ion mode, source voltage 5 kV, capillary voltage −48 V, tube lens voltage −176.47 V. Capillary temperature for both positive and negative ion modes was 280 °C. MS spectra were acquired by full range acquisition covering *m*/*z* 150–1000.

Data were acquired using Xcalibur software version 2.1, and for fragmentation studies, a data dependent scan experiment was carried out selecting precursor ions as the most intensive peaks in the LC-MS analysis.

Identification of compounds was based on retention times, accurate mass measurements, MS/MS data, exploration of specific spectral libraries and public repositories for MS-based metabolomic analysis (*MassBank MoNA*, *MassBank NORMAN*, *PubChem*), and comparison with data reported in the literature [12,13,14,15,16,17,18,19,20,21,22,23,24].

### 2.4. Oxygen Radical Absorbance Capacity (ORAC) Assay

According to Moudache et al. [9], 25 μL of different concentrations of (0.01–0.2 mg/mL) CAE were incubated in a 96-well microplate with 125 μL of fluorescein (10 nM in 75 mM NaH_2_PO_4_ buffer at pH 7.4) for 30 min at 37 °C. Then, 25 μL of 10 mM AAPH was added to each well and fluorescence was recorded (λ_ex_ 485 nm and λ_em_ 520 nm) every 2 min for 90 min using a GLOMAX Multidetection System (Promega, Madison, WI, USA). Trolox (0–100 μM) was used as the reference standard. Results were calculated on the basis of differences in areas under the fluorescence decay curve between the blank, samples, and standards. Final oxygen radical absorbance capacity (ORAC) values were expressed as μmol of Trolox equivalents (TE)/100 g of dried extract (DE).

### 2.5. Liposome Preparation and Characterization

For the preparation of liposomes, 90 mg/mL of P90G and 2 mg/mL of CAE were weighed in a glass vial, dispersed in water, and sonicated (25 cycles, 5 s on and 2 s off; 13 μm of probe amplitude) with a high intensity ultrasonic disintegrator (Soniprep 150, MSE Crowley, London, UK).

Empty liposomes (i.e., without extract) were prepared under the same conditions as extract-loaded liposomes.

The average diameter (i.e., the intensity weighed mean hydrodynamic size), polydispersity index (P.I., a dimensionless measure of the broadness of the size distribution), and zeta potential of the liposomes were determined by dynamic and electrophoretic light scattering using a Zetasizer nano-ZS (Malvern Instruments, Worcestershire, UK). Samples (*n* > 10) were diluted with bidistilled water (1:100 *w*/*v*) and analyzed at 25 °C.

The stability of liposomes was evaluated by long-term stability tests, i.e., by analyzing vesicle average diameter, P.I., and zeta potential over two months at 25 °C.

### 2.6. Cell Line and Culture Conditions

Human hepatocellular carcinoma cell line (HepG2) cells were cultured in DMEM (supplemented with 10% fetal bovine serum, 2 mM glutamine, 100 U/mL penicillin, and 100 μg/mL streptomycin) and maintained at 37 °C in a humidified atmosphere containing 5% CO_2_. The CAE was dissolved in EtOH/DMSO and different concentrations were tested (10–200 µg/mL). EtOH/DMSO-treated cells were used as the control (CTRL) in all the experiments.

### 2.7. MTT Assay

Cell viability was evaluated on HepG2 cells by the MTT assay, a colorimetric assay based on the conversion of the yellow tetrazolium salt MTT into purple insoluble formazan by the succinate dehydrogenase enzyme of viable cells. HepG2 cells were seeded in a 96-well plate (1.5 × 10^4^ cells/well), incubated overnight, and treated with different concentrations (10–200 µg/mL) for 24 and 48 h. After removal of the medium, the cells were washed with PBS and incubated with 0.75 mg/mL of MTT solution in PBS for 4 h. Then, the solution was removed and the cells were lysed using a solubilization solution (1:1 DMSO:isopropanol). The solubilized formazan product was spectrophotometrically quantified at 560 nm using a UV–Vis spectrophotometer (SPECTROstar^Nano^ BMG Labtech, Ortenberg, Germany).

### 2.8. Measurement of Intracellular Reactive Oxygen Species (ROS)

The intracellular reactive oxygen species (ROS) level was measured by DCFH-DA [25]. HepG2 cells were plated at a density of 1 × 10^4^ cells/well in a 24-well plate, incubated with different concentrations of CAE (10–200 µg/mL), liposomes, or 10 mM NAC for 24 h, and stressed with 5 mM *t*-BuOOH for 1 h. Finally, the cells were stained with 10 µM DCFH-DA for 30 min at 37 °C in the dark, and fluorescence was measured by BD FACSCanto II (BD Pharmingen, San Jose, CA, USA) (λ_ex_ 485 nm and λ_em_ 515–540 nm).

### 2.9. Quantitative RT-PCR

HepG2 cells were treated with different concentrations of CAE (200–100 µg/mL) for 24 h. RNA was extracted using Quick-RNA MiniPrep kit (Zymo Research, Irvine, CA, USA) and then was transcribed to cDNA using random primers and a High-Capacity cDNA Reverse Transcription Kit (ThermoFisher scientific, Waltham, MA, USA, Life Technologies Corporation, Carlsbad, CA USA). The cDNA was amplified via real-time PCR using iTaqTM Universal SYBR^®^ Green Supermix (Bio-Rad) by the 7500 Fast Real-Time PCR System (Applied Biosystems, Foster City, CA, USA). Primers were designed for spanning exon–exon junctions, eliminating undesirable genomic DNA amplification. The comparative threshold cycle method (ΔΔCt) was used to quantify the relative amounts of product transcripts with *β*-actin as the housekeeping gene [26,27]. The specificity of amplicons was confirmed by melting-curve analysis. Each test was performed in triplicate.

### 2.10. Statistical Analysis

Data were expressed as mean ± standard deviation (Mean ± SD). Statistical analysis was performed using GraphPad Prism 5 Software, Inc. (San Diego, CA, USA) and *p* values ≤ 0.05 were considered as statistically significant.

## 3. Results

### 3.1. Phytochemical Profile of C. annuum Extract

Qualitative analysis of CAE was performed by LC-ESI-Orbitrap-MS and LC-ESI-Orbitrap-MS/MS analyses. The use of the Luna C18 column and LC-ESI-MS/MS (alternating positive and negative ionization modes) allowed for the simultaneous separation and identification of all compounds (non-polar and polar compounds) in the ethanol extract. With a short interscan delay of 0.3 s, a number of scans in each mode across each chromatographic peak were obtained.

**Figure 1:**
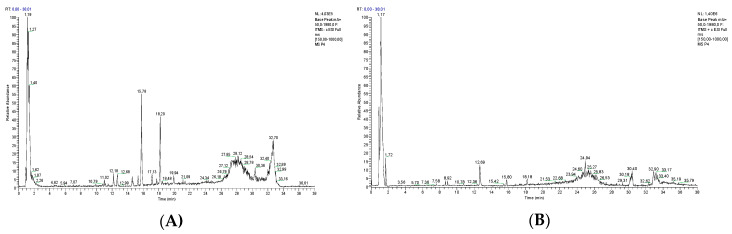
LC-MS profile of *C. annuum* ethanol extract in negative (**A**) and positive (**B**) ion mode.

This procedure allowed us to save time and use a lower amount of sample, because only one injection positive and negative ion data were recorded. The developed LC-ESI-MS/MS method can easily be utilized as a fast and sensitive analytical tool for analysis of phytochemical profiles of Senise pepper. Data are showed in Figure 1. Individual components were identified by comparison of their *m*/*z* values in the total ion current (TIC) profile with those of the selected compounds described in the literature. In particular, 24 compounds were identified in CAE (Table 1) belonging to a wide variety of structurally different metabolic classes: phenols (caffeic acid and 2,4-di-*tert*-butylphenol); capsinoids (capsiate and dihydrocapsiate); carotenoids (*β*-carotene, capsorubin, antheraxanthin, and *β*-cryptoxanthin); sesquiterpenoids (canusesnol F); flavones (luteolin, luteolin-apiosylacetyl–glucoside, apigenin-6,8-di-*C*-glucoside, and vitexin); flavonols (isoquercetin, rutin, kaempferol-3-*O*-glucoside, kaempherol, and myricetin); flavan-3-ols (catechin); vitamins (ascorbic acid and tocopherol); and capsaicinoids (nordihydrocapsaicin, capsaicin, and dihydrocapsaicin).

### 3.2. Antioxidant Activity: ORAC Assay

**Figure 2:**
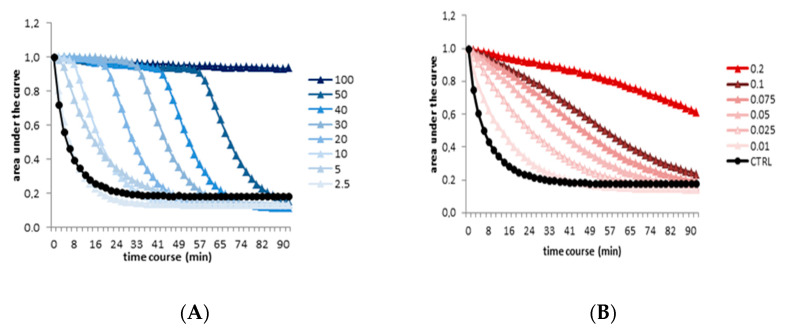
(**A**) Oxygen radical absorbance capacity (ORAC) assay for different concentrations (2.5–100 µM) of Trolox used as a standard. (**B**) ORAC assay for different concentrations (0.01–0.2 mg/mL) of CAE. Changes in the fluorescence intensity of fluorescein were monitored for 90 min.

The antioxidant activity of CAE was evaluated by the ORAC assay. As shown in Figure 2, the extract slowed fluorescein degradation by quenching the peroxyl radicals in a dose-dependent manner. An ORAC value of 38,144 μmol TE/100 g of DE was obtained by using the Trolox standard curve (y = 0.48x − 3.38; R^2^ = 0.99).

### 3.3. Liposome Characterization

Liposomes were prepared by a simple, organic, solvent-free method involving the sonication of a phospholipid (P90G) and CAE in water. To evaluate the effect of the incorporation of the extract into the vesicle arrangement, empty liposomes (i.e., without extract) were also prepared and characterized.

Light scattering results, summarized in Table 2, showed that empty liposomes were small in size, around 80 nm, with good homogeneity (P.I. 0.28) and negative zeta potential (∼−17 mV). The incorporation of CAE did not alter these values (*p* > 0.05), which points to a negligible effect of the extract on the vesicle assembly.

It is well known that the stability of liposomes is dependent on both formulation and manufacturing method parameters, and it is critical to establish safe and effective use of the liposomes. Therefore, the stability of the prepared liposomes was evaluated by monitoring the size, P.I., and zeta potential over two months of storage. The results showed no significant variations (*p* > 0.05) of the parameters examined, which indicates a good stability of the vesicle formulations.

### 3.4. Effect of C. annuum Extract on Cell Viability and Intracellular ROS

**Figure 3:**
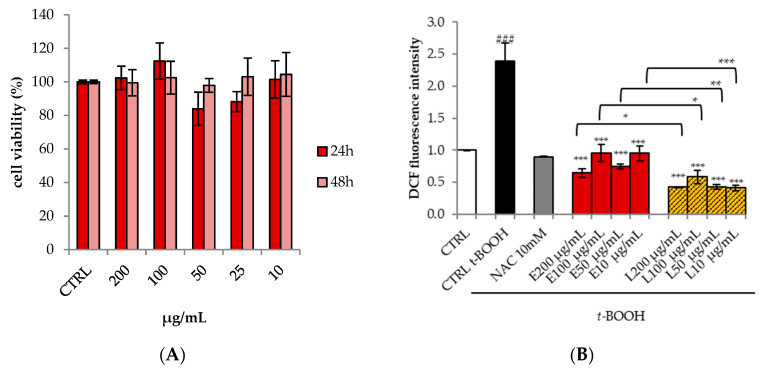
(**A**) Cell viability, evaluated by MTT assay, of HepG2 cells treated for 24 and 48 h with different concentrations of *C. annuum* extract (CAE). Data are expressed as the mean ± SD of three independent experiments (*n* = 3). (**B**) Effects of CAE (E) and liposomes (L) on *t*-BuOOH-induced intracellular reactive oxygen species (ROS) generation in HepG2 cells. Cells were pre-treated with the extracts or liposomes at different concentrations (10, 25, 50, 100, 200 μg/mL) for 24 h and subsequently incubated with 5 mM *t*-BuOOH for 1 h. ROS generation was measured by DCFH-DA staining with flow cytometry analysis. Data are expressed as the mean ± SD of three independent experiments (*n* = 3). ^###^ *p* < 0.001 vs. CTRL, *** *p* < 0.001 vs. *t*-BuOOH-treated cells, * *p* < 0.05, ** *p* < 0.01.

The cell viability of CAE was evaluated on the HepG2 cell line, used as model cells, by the MTT assay. The extract was dissolved in EtOH/DMSO and the final concentrations (1.6% and 0.4%, respectively) of solvent used for HepG2 cell assays had no effect on cell viability. As shown in Figure 3A, the extract showed no cytotoxic effect after 24 and 48 h.

The protective effect of the extract against intracellular ROS was also investigated (Figure 3B). HepG2 cells were treated with different concentrations of extract (10–200 μg/mL) for 24 h and then oxidative stress was induced by *t*-BuOOH, known as a source of ROS. HepG2 cells stressed with *t*-BuOOH showed a 2-fold increase in fluorescence, as compared to untreated cells. The pre-treatment with the extract for 24 h reduced ROS levels dramatically, restoring the basal level similar to that of cells treated with NAC, a known antioxidant.

CAE was incorporated in liposomes to evaluate the effect of the formulation on its biological activity. More specifically, the protective effect of extract-loaded liposomes was evaluated in *t*-BuOOH–stressed HepG2 cells after 24 h of treatment (Figure 3B). The liposomes were tested at the same concentrations of the raw extract (10–200 μg/mL). It is interesting to note that when the extract was incorporated into liposomes, the ROS levels were significantly decreased (by 6 times vs. *t*-BuOOH control cells), already at 10 μg/mL, the lowest concentration tested, thus being twice as potent in comparison with both the raw extract and NAC (Figure 3B).

### 3.5. Effect of C. annuum Extract on Gene Expression

**Figure 4:**
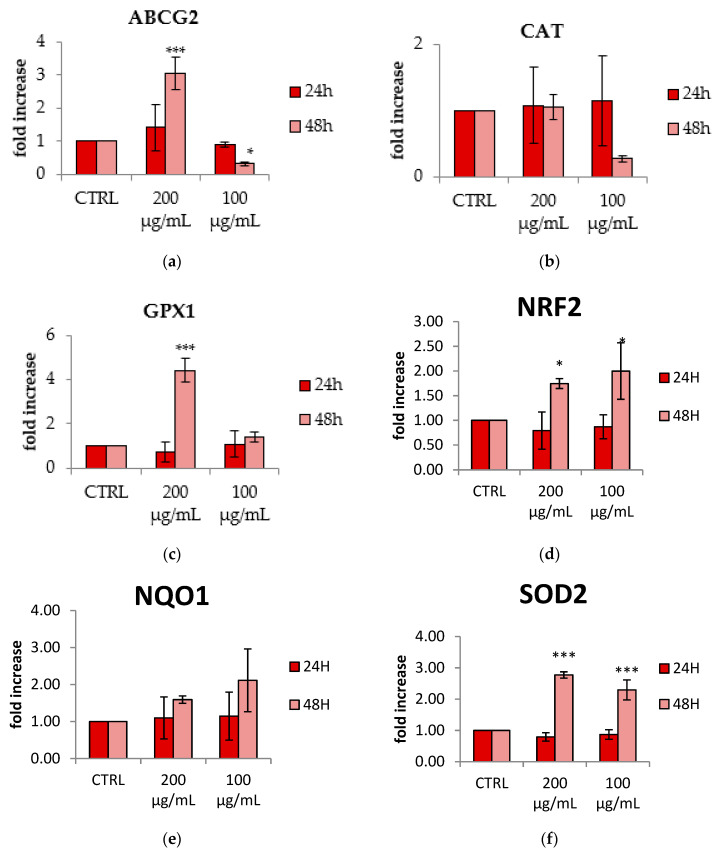
Effect of CAE (200 and 100 μg/mL) on the gene expression of (**a**) ATP-binding cassette transporter G2 ABCG2, (**b**) catalase (CAT), (**c**) glutathione peroxidase (GPx-1), (**d**) nuclear factor erythroid 2-related factor 2 (Nrf-2), (**e**) NADPH-quinone oxidase (NQO1), (**f**) superoxide dismutase (SOD-2) analyzed by real-time q-PCR and normalized with the housekeeping gene, actin, in HepG2 cell line. Data are expressed as mean ± SD of three independent experiments (*n* = 3). * *p* < 0.05, *** *p* < 0.001 vs. control (CTRL).

HepG2 cells were treated with CAE (200 and 100 μg/mL) and the expression of some genes involved in antioxidant defense was evaluated by qRT-PCR. After 24 h of treatment, the extract did not affect the expression of genes, as compared to the control (CTRL) (Figure 4). In contrast, the extract upregulated the expression of SOD-2 and GPx-1 after 48 h, as well as the nuclear factor erythroid 2-related factor 2 (Nrf2) and ATP-binding cassette transporter G2 (ABCG2) (Figure 4). No statistical differences were found in the expression of catalase (CAT) and NADPH-quinone oxidase 1 (NQO1) (Figure 4).

## 4. Discussion

Fruits of *C. annuum* cv Senise were extracted by exhaustive maceration with absolute ethanol achieving an extraction yield of 11.70% *w*/*w*, similar to that reported by Loizzo et al. who showed that ethanol improved the yield of pepper extract compared to exane (9.8 ± 0.8% vs. 0.5 ± 0.06%) [7].

The potential health benefits of *C. annuum* L. cv Senise dried pepper was demonstrated previously by Loizzo et al. [7]. In particular, the authors demonstrated the good radical scavenging activity of an ethanol extract against two synthetic radicals, DPPH and ABTS (IC_50_ = 55.0 ± 1.8 μg/mL, TEAC value = 12.6 ± 1.1, respectively) [7]. In the present study, the antioxidant activity of CAE extract was confirmed, reporting an ORAC value of 38,144 μmol TE/100 g of dried extract. The result was comparable with other cultivars of red sweet pepper [28] and other known antioxidant foods, such as *Olea europeae* L. (ORAC value of leaf extract = 69,639 µmol TE/100 g of dried weight) [28]. To the best of our knowledge, this is the first work that reports the biological activity of *C. annuum* cv Senise extract in cells. The extract was demonstrated to protect cells from oxidative stress, reducing intracellular ROS, by activating transcription factors that induce the expression of antioxidant enzymes. These beneficial effects could be ascribed to the presence of health-promoting compounds that were evaluated for the first time in Senise dried pepper. In particular, qualitative analysis performed with LC-MS/MS identified 24 compounds belonging to polyphenols, carotenoids, capsinoids, and vitamins.

Many studies have reported that polyphenols and carotenoids promote the expression of Nrf-2 [4], a redox sensitive transcription factor that provokes an antioxidant response by inducing the expression of cytoprotective enzymes, such as glutathione *S*-transferase (GST), superoxide dismutase (SOD), heme oxigenase-1 HO-1, and NADPH-quinone oxidase NQO1 [29,30,31]. The cells treated with CAE showed a higher level of Nrf-2, as well as of SOD-2 and Gpx-1 expression, suggesting that the extract exerts its antioxidant activity by acting through this pathway. Previous studies evidenced that Nrf-2 expression also influences the expression of ABCG2, a transmembrane transporter protein responsible of GSH transport [11,31,32] and associated with redox regulation in some pathological conditions such as Alzheimer’s and cardiovascular diseases [33,34,35,36]. In our study, both Nrf-2 and ABCG2 increased after 48 h of treatment with the extract. Moreover, the polyphenols present in the extract can interact directly with this transporter, as reported in previous studies [37].

In addition, the extract was incorporated into liposomes to explore their potential in favoring the internalization in cells, with a consequent enhancement of the efficacy of the extract. Liposomes have been demonstrated to increase the solubility and stability of polyphenols and carotenoids, which results in improved bioavailability and therapeutic benefit [5,6]. As reported by Caddeo et al. [38], cellular uptake of quercetin and resveratrol improved when they were incorporated in liposomes, ameliorating the biological activity in vitro and in vivo. In this study, liposomes were found to improve the antioxidant activity of *C. annuum* extract. More specifically, the cells treated with extract-loaded liposomes showed lower levels of ROS than cells treated with the extract, with liposomes being twice as active in comparison with the raw extract at equal concentrations. This means that the same beneficial effect, or even a stronger one, can be achieved with a lower dose, thus reducing costs and possible side effects. Probably this is due to a higher quantity of compounds that reach the cells when the extract is encapsulated into the liposomes, but further investigations are needed to understand the mechanistic aspects related to the measured CAE biological effects.

## 5. Conclusions

In this study, the effects of ethanolic extract of *Capsicum annuum* L. cv Senise were studied with particular regard to gene expression and ROS generation in cell models. It is clear that several genes involved in the redox cell system are activated during treatment with the extract, with an evident impact on SOD-2 and GPX-1 as well as Nrf2 and ABCG2. Overall, this study suggests that a typical food of the Basilicata region could represent a new strategy in nutraceutical and pharmaceutical fields. For the first time, the protective effect of *Capsicum annuum* L. cv Senise against oxidative stress was investigated in cells, and twenty-four compounds were identified with LC-MS. Results obtained demonstrated the activity of CAE, but further experiments are needed to understand if some of the identified compounds are responsible for the measured synergist effect. Moreover, interesting results were obtained by formulating the extract into liposomes, which potentiated its antioxidant activity. These findings support the association of antioxidants of natural origin with nanocarriers to develop health-promoting systems.

## Figures and Tables

**Table 1:** Metabolites identified in *C. annuum* ethanol extract using LC-ESI/Orbitrap/MS/MS.

Compounds	Rt (min)	Molecular Formula	MW	[M − H]^−^	[M − H]^+^	MS/MS
Caffeic acid	1.19	C_9_H_8_O_4_	180.15	179		135,174
Luteolin (apiosyl acetyl) glucoside	1.33	C_20_H_30_O_16_	621.14	620		327
Apigenin-6,8-di-C-glucoside	1.99	C_27_H_30_O_15_	594.15	593		473
Vitexin	2.34	C_21_H_20_O_10_	432.38	431		283,311
Isoquercetin	4.95	C_21_H_20_O_12_	464.09	463		301
Rutin	5.64	C_27_H_30_O_16_	610.52	609		225,387
Kaempferol-3-*O*-glucoside	6.01	C_21_H_20_O_11_	448.38	447		285
Catechin	33.08	C_15_H_14_O_6_	290.21	289		203
2,4-Di-tert-butylphenol	33.10	C_14_H_22_O	206.32	205		189
Capsiate	33.11	C_18_H_26_O_4_	306.40	305		151,289
Ascorbic acid	33.14	C_6_H_8_O_6_	176.12	175		112
Dihydrocapsiate	1.10	C_18_H_28_O_5_	308.40		309	278,295
Luteolin	1.29	C_15_H_10_O_6_	286.24		287	153,171.2
Kaempherol	1.39	C_15_H_10_O_6_	286.23		287	241
Nordihydrocapsaicin	1.72	C_17_H_27_NO_3_	293.41		294	152
Tocopherol	7.80	C_29_H_50_O_2_	430.71		431	416
Myricetin	12.53	C_15_H_10_O_8_	318.23		319	227,207
Capsaicin	15.56	C_18_H_27_NO_3_	305.41		306	137,227
Dihydrocapsaicin	17.43	C_18_H_29_NO_3_	307.43		308	122,207
*β*-carotene	18.22	C_40_H_56_	536.87		537	277,353
Canusesnol F	19.47	C_15_H_22_O_4_	266.33		267	207,247
Capsorubin	26.62	C_40_H_56_O_4_	600.88		601	411,582
Antheraxanthin	27.77	C_40_H_56_O_3_	584.88		585	145
*β*-cryptoxanthin	32.98	C_40_H_56_O	552.88		553	461

**Table 2:** Characteristics of empty liposomes and *C. annuum*-loaded liposomes: intensity-weighed mean hydrodynamic diameter, polydispersity index (P.I.), and zeta potential.

	Mean Diameter (nm)	P.I. ^#^	Zeta Potential (mV)
Empty liposomes	81.6 ± 6.8	0.28	−16.7 ± 3.7
*C. annuum* liposomes	83.8 ± 4.7	0.26	−17.5 ± 4.2

Each value represents the mean ± SD, *n* > 10; ^#^ SD for P.I. values was always <0.03.
